# Genomic and metabolic uniformity across diverse observed ecological contexts suggest that interactions of *Pestalotiopsis formosana* and *P. neolitseae* are context-dependent

**DOI:** 10.3897/imafungus.17.192779

**Published:** 2026-05-28

**Authors:** Sheng-Yu Hsu, Bagdevi Mishra, Hsin-Yao Huang, Yu-Chen Lin, Yuan-Cheng Xu, Sebastian Ploch, Chia-Ying Hsu, Jau-Ping Wang, Abdallah M. Elgorban, Khaloud M. Alarjani, Marco Thines, Hiran A. Ariyawansa

**Affiliations:** 1 Department of Plant Pathology and Microbiology, National Taiwan University, 106319, Taipei, Taiwan Senckenberg Biodiversity and Climate Research Centre Frankfurt Am Main Germany; 2 Senckenberg Biodiversity and Climate Research Centre, Senckenberganlage 25, 60325, Frankfurt Am Main, Germany High-Value Food from Mushrooms and Bioactive Plants in the Green Economy Value Chain Research Group, The Institute of Biotechnology and Genetic Engineering, Chulalongkorn University Bangkok Thailand; 3 Center of Excellence in Biotechnology Research (CEBR), King Saud University, 12372, Riyadh, Saudi Arabia Center of Excellence in Bioconversion and Bioseparation for Platform Chemical Production, The Institute of Biotechnology and Genetic Engineering, Chulalongkorn University Bangkok Thailand; 4 Department of Botany and Microbiology, College of Science, King Saud University, Riyadh, 11451, Saudi Arabia Center of Excellence in Biotechnology Research (CEBR), King Saud University Riyadh Saudi Arabia; 5 Department of Biological Sciences, Institute of Ecology, Evolution and Diversity, Goethe University, Max-Von-Laue-Straße 13, 60483, Frankfurt Am Main, Germany Department of Botany and Microbiology, College of Science, King Saud University Riyadh Saudi Arabia; 6 Center of Excellence in Bioconversion and Bioseparation for Platform Chemical Production, The Institute of Biotechnology and Genetic Engineering, Chulalongkorn University, 254 Phayathai Road, Pathumwan, Bangkok, 10330, Thailand Department of Plant Pathology and Microbiology, National Taiwan University Taipei Taiwan; 7 High-Value Food from Mushrooms and Bioactive Plants in the Green Economy Value Chain Research Group, The Institute of Biotechnology and Genetic Engineering, Chulalongkorn University, 254 Phayathai Road, Pathumwan, Bangkok, 10330, Thailand Department of Biological Sciences, Institute of Ecology, Evolution and Diversity, Goethe University Frankfurt Am Main Germany

**Keywords:** Carbon-use profiles, CAZymes, comparative genomics, ecological flexibility, lifestyle differentiation, pestalotioid fungi, plant-associated fungi

## Abstract

Members of the genus *Pestalotiopsis* have been reported from symptomatic, asymptomatic, and dead plant tissues, and are therefore frequently described as phytopathogens, endophytes, or saprobes. This study focused on two closely related species, *Pestalotiopsis formosana* and *P. neolitseae*, which have been isolated from saprobic, endophytic, and pathogenic contexts in recent studies, raising questions regarding the basis for these diverse observed lifestyles. To clarify this ambiguity, we sequenced six new genomes representing strains of these species spanning these observed lifestyles and compared them with four publicly available, curated plant-associated genomes. Despite contrasting field observations, strains of *P. formosana* and *P. neolitseae* showed nearly identical genome features, sharing a core genome of 12,021 orthologous proteins with almost identical secretome content, effectors, CAZyme repertoires, and secondary metabolite gene clusters. Carbon-use assays (Biolog FF and minimal media) further showed broadly overlapping metabolic capabilities, although some strain-level differences were observed. CAZyme-based trophic prediction (CATAStrophy) also placed all analysed strains within the same broad trophic prediction space. Taken together, these results do not support clear genome-scale differentiation corresponding to the assigned lifestyle categories within the present sampling framework. Instead, the data are consistent with the interpretation that these fungi share a broadly conserved genomic toolkit, while ecological expression may depend on regulatory, physiological, host-related, and environmental factors. These findings provide a comparative framework for future studies integrating transcriptomics, metabolomics, and experimental infection assays to clarify how ecological behaviour is expressed in *Pestalotiopsis*.

## Introduction

Fungi have adopted diverse lifestyles such as symbionts, commensals, or pathogens across a broad range of hosts (Giraud et al. 2010). Lifestyle-associated adaptations have received particular attention because the emergence of a pathogen often involves a notable increase in virulence from a previously weak pathogen (Giraud et al. 2010; Ohm et al. 2012; Kabbage et al. 2015; Haridas et al. 2020).

Several studies have explored the common genetic signatures of fungal species exhibiting diverse lifestyles and identified unique characteristics associated with each lifestyle. Such efforts have focused on genera such as *Colletotrichum* (Gan et al. 2013; Hacquard et al. 2016), *Cryphonectria* (Stauber et al. 2020), *Fusarium* (Hill et al. 2022; Ulaszewski et al. 2025), and *Phyllosticta* (Buijs et al. 2022). By comparing species with different lifestyles within a given genus, significant variation in the abundance of CAZymes, secondary metabolites (SMs), and effectors were found. For instance, Gan et al. (2013) found that mutualistic symbionts and biotrophic pathogens of *Colletotrichum* possess more species-specific secreted proteins (pathogenicity effectors) compared to saprotrophs and necrotrophic pathogens. In contrast, Hill et al. (2022) reported no major differences between phytopathogenic and endophytic fusarioid fungi regarding effectors, CAZymes, or gene repertoires. However, a study including a broader taxonomic range of *Nectriaceae* could find such differences for various fusarioid genera, even though within *Fusarium* no clear-cut differences were found (Ulaszewski et al. 2025). It is noteworthy, that all of these studies have focussed on rather global patterns, not specifically addressing the within-species diversification according to observed lifestyles.

In a recent study of *Cryphonectria*, Stauber et al. (2020) concluded that the loss of certain CAZymes may have promoted the pathogenicity of *Cryphonectria parasitica* (which causes disease in *Castanea* spp.) compared to non-pathogenic *Cryphonectria* species. Stauber et al. (2020) also noted that putative effectors differed substantially in number, cysteine-rich gene content, and protein length among taxa, whereas secondary metabolite gene clusters remained largely conserved. Likewise, during investigations into *Phyllosticta*, Buijs et al. (2022) identified several genomic differences among species, including unique gene clusters found only in pathogens or only in endophytes. They further showed that CAZymes profiles clustered independently of phylogenetic relationships, as demonstrated by the CATAStrophy tool (Hane et al. 2020). Notably, Buijs et al. (2022) clarified the ambiguous lifestyle of *Phyllosticta citrichinaensis*, initially classified as a weak pathogen, by revealing genomic and phenotypic traits characteristic of both pathogens and endophytes, indicating an intermediate lifestyle. However, in some cases, predicting CAZyme content from DNA-based genomic methods provides limited insight into the natural growth status of fungi (Mäkelä et al. 2020).

Members of the genus *Pestalotiopsis* are widely distributed in tropical and temperate regions and exhibit highly diverse lifestyles (Bate-Smith and Metcalfe 1957; Lv et al. 2011; Monden et al. 2013; Maharachchikumbura et al. 2014; Reddy et al. 2016; Ariyawansa and Hyde 2018; Zhang et al. 2021). Notably, *Pestalotiopsis* species have repeatedly been described as endophytes, saprobes, and aggressive pathogens, sometimes even within the same species, suggesting a remarkable degree of ecological flexibility. (Maharachchikumbura et al. 2012; Xu et al. 2014). A large number of *Pestalotiopsis* species have been reported as plant pathogens, capable of causing a variety of diseases across numerous hosts (Maharachchikumbura et al. 2014; Luo et al. 2024). Notably, in recent years, *Pestalotiopsis* species have been identified as emerging plant pathogens. For example, *Pestalotiopsis pini* has emerged as a primary pathogen causing shoot blight and trunk necrosis in *Pinus pinea* in the Mediterranean (Silva et al. 2020). In Spain, 42.68% of fungal diseases affecting *Pinus* species in 2022 were identified as *Pestalotiopsis* (Monteiro et al. 2022). It has been suggested that species in the genus can switch nutritional modes, raising the question of whether they have undergone a lifestyle shift to become primary pathogens due to external factors or adaptation to a primarily pathogenic lifestyle (Maharachchikumbura et al. 2014).

Several studies have examined the genomes of *Pestalotiopsis* species to elucidate their disease mechanisms or colonization strategies. For example, Yang et al. (2023) investigated the phytopathogenic *P. neglecta* YJ-3, which causes black spot needle blight on *Pinus sylvestris* var. *mongolica*. Their analysis suggested that the abundance of glycoside hydrolases (GH) and glycosyltransferases (GTs) in the genome could facilitate plant cell wall degradation during infection. Additionally, Wang et al. (2015) studied the endophytic strain *Pestalotiopsis fici* W106-1 (isolated from tea plants) and found it had more pectinase-encoding genes than several well-known plant pathogens such as *Fusarium graminearum*, *F. verticillioides*, *F. oxysporum*, *Magnaporthe oryzae*, and *Sclerotinia sclerotiorum*, which may help it adapt to an endophytic lifestyle. However, these conclusions are based on individual genome analyses or comparisons with distantly related fungi.

In a previous study, two novel *Pestalotiopsis* species from Taiwan, *P. formosana* and P*. neolitseae*, were described (Ariyawansa and Hyde 2018). Initially, *P. formosana* was recovered from dead grasses, whereas *P. neolitseae* was associated with leaf spots on *Neolitsea villosa* (Ariyawansa and Hyde 2018). Subsequently, both species were also reported in association with tip blight of *Podocarpus costalis*, and a newly described pathogenic species, *P. sonneratiae* NTUPPMCC 21-044, was identified as a close relative of both taxa (Xu et al. 2025). In the present study, we also recovered *P. formosana* NTUPPMCC 22-221 from surface-sterilized asymptomatic *Podocarpus* leaves. These contrasting reports raise the question of whether isolates recovered from different ecological contexts show detectable genomic differentiation, or whether they reflect overlapping ecological potential expressed under different conditions. To address this question, we sequenced, assembled, and annotated six new genomes representing isolates recovered from symptomatic, asymptomatic, and dead plant tissues of *P. formosana* and *P. neolitseae*, and compared them with four publicly available genomes. Through integrative comparative analyses of orthologs, secretomes, effectors, CAZymes, secondary metabolites, transposable elements, and carbon-use profiles, we evaluated whether reported lifestyle differences correspond to distinct genomic signatures or whether these species represent inherently plastic vasculartrophic fungi whose lifestyle expressions depend on context and environmental conditions.

## Materials and methods

### Identification of the correct taxonomic position of Pestalotiopsis genomes in GenBank

In addition to the genomes generated in the present study, *Pestalotiopsis* genomes from the NCBI genome database were used for comparative analysis. However, the correct taxonomy and phylogenetic relationships of these publicly available genomes, including those of *Pestalotiopsis*, need careful revision, as recently outlined by Steenwyk et al. (2023). At the beginning of this study, 11 genomes of *Pestalotiopsis* strains were available in the NCBI GenBank (https://www.ncbi.nlm.nih.gov/datasets/genome/, data accessed on 11 June 2024). Because our primary aim was to compare isolates of *P. formosana* and *P. neolitseae* recovered from contrasting plant contexts, preliminary phylogenetic checks were necessary to ensure that all comparative genomes belonged to *Pestalotiopsis* sensu stricto and were taxonomically reliable. These analyses confirmed that *Pestalotiopsis fici* W106-1 clusters within *Pseudopestalotiopsis*, and this strain was therefore retained only as an outgroup taxon. Additionally, this study excluded strains that were either unrelated to plant habitats or had unclear ecological metadata. Lifestyle designations were treated as operational labels based on the source of isolation and host symptomatology. i.e. they correspond to observed lifestyles and contexts at the time of isolation. Specifically, for strains sequenced in this study, isolates recovered from surface-sterilized, asymptomatic plant tissues were described as putatively endophytic (E) (Keim et al. 2014), whereas those recovered from active disease lesions were described as putatively pathogenic (PP). For the latter, pathogenicity was further supported by mycelial-plug inoculation assays conducted on the original host tissues (Ariyawansa and Hyde 2018; Xu et al. 2025), even though these assays need to be interpreted as evidence of pathogenic potential under controlled conditions rather than definitive proof of general ecological behaviour under natural conditions. Isolates obtained from dead or decaying plant material were described as putatively saprobic (S), following the common practice in ecological studies without denying the possibility that the observed context might not represent the entire ecological niche of the isolates. For publicly available genomes, ecological designations were adopted from the associated NCBI metadata and source publications when available. The ecological designations and habitats of the final *Pestalotiopsis* strains included in this study are provided in Table 1 and Suppl. material 1: table SS2.

**Table 1.** The details of selected strains used in comparative genomic analysis.

Taxon	Strain	Host/habitat	Observed ecological context	Geographic origin	Sampling date	Reference
*P. camelliae-oleiferae*	NC0098	*Acer rubrum*	E	Highlands, North Carolina, USA	01-Mar-2022	Franco et al. (2022)
*P. clavata*	JCM9685	*Taxus brevifolia*	E	Bozeman, MT, USA	1995	Direct submission
	YJ-3	*Pinus sylvestris* var. *mongolica*	PP	Hulunbuir, China	Aug-2020	Yang et al. (2023)
***P. formosana***	**NTUCC 17-009 (= BG011)**	**Grass (*Poaceae*)**	**S**	**Taipei Botanical Garden, Taipei City, Taiwan**	**09-Aug-2017**	**Ariyawansa and Hyde (2018)**
	**NTUPPMCC 21-035 (= SCL-4)**	***Podocarpus costalis***	**PP**	**Shanchong Village, Zhushan Township, Nantou County, Taiwan**	**27-Nov-2021**	**Xu et al. (2025)**
	**NTUPPMCC 22-221 (= TUL-4)**	***Podocarpus costalis***	**E**	**National Taiwan University, Taipei City, Taiwan**	**26-Aug-2022**	**This study**
*P. hainanensis*	9143b	*Platycladus orientalis*	E	Duke Forest, North Carolina, USA	Jun-2006	Shaffer et al. (2022)
***P. neolitseae***	**NTUCC 17-011 (= BG002)**	***Neolitsea villosa***	**PP**	**Taipei Botanical Garden, Taipei City, Taiwan**	**09-Aug-2017**	**Ariyawansa and Hyde (2018)**
	**NTUPPMCC 21-036 (= SCL-2)**	***Podocarpus costalis***	**PP**	**Shanchong Village, Zhushan Township, Nantou County, Taiwan**	**27-Nov-2021**	**Xu et al. (2025)**
***P. sonneratiae***	**NTUPPMCC 21-044 (= ZSL-4)**	***Podocarpus costalis***	**PP**	**Zhushan Village, Zhushan Township, Nantou County, Taiwan**	**27-Nov-2021**	**Xu et al. (2025)**
*Ps. fici*	W106-1	*Camellia sinensis*	E	Hangzhou, China	10-Jan-2014	Wang et al. (2015)

Notes: *P*, *Pestalotiopsis*; *Ps*, *Pseudopestalotiopsis*; E, isolate recovered from asymptomatic tissue; PP, isolate recovered from symptomatic tissue; S, isolate collected from dead or decaying material; Newly sequenced genomes are indicated in bold.

The current classification of *Pestalotiopsis* species is primarily based on phylogenetic relationships derived from DNA sequence data of the ITS, tub2, and tef1-α loci (Maharachchikumbura et al. 2014). To incorporate strains with available whole-genome sequences (WGS) into our phylogenetic analysis, we first extracted these marker genes from the WGS data. Briefly, the *Pestalotiopsis* genome FASTA files downloaded from the NCBI database were used to construct local nucleotide databases using the makeblastdb command in NCBI-BLAST+ (v2.12.0+ds-3build1) (Camacho et al. 2009). Reference ITS, tub2, and tef1-α sequences of *Pestalotiopsis* species obtained from recent literature (detailed in Suppl. material 1: table SS1) were then used as queries to search against these local genome databases using BLASTn. The BLAST output was formatted (-outfmt “6 qseqid sseqid sallseqid score evalue sstart send”) to identify the genomic coordinates of the target loci. Based on these coordinates, the ITS, tub2, and tef1-α sequences were extracted from the WGS assemblies. Finally, the newly extracted WGS marker sequences were combined with the downloaded reference sequences (table SS1) to generate the final concatenated multi-locus sequence matrix for downstream alignment and phylogenetic tree construction.

After obtaining information on the sequence locations in the genomes, sequences spanning 500 base pairs upstream and downstream of the target sequences were extracted using a Python script. Once the relevant ITS, *tub2*, and *tef1-α* sequences were identified, multiple sequence alignments were generated using MAFFT v.7 (http://mafft.cbrc.jp/alignment/server/index.html) with default parameters. To determine the phylogenetic placement of these taxa, phylogenetic trees were first constructed separately for ITS, *tub2*, and *tef1-α*. The sequences from these loci were then concatenated for a multi-locus analysis using the Maximum Likelihood (ML) approach. The best-fit substitution models (ITS: K2P+I+G4, *tub2*: K2P+I+G4, *tef1-α*: TNe+G4) were selected for each locus according to the Akaike Information Criterion (AIC), based on a Nexus-formatted partition file generated by the IQ-TREE web server (http://iqtree.cibiv.univie.ac.at/) (Trifinopoulos et al. 2016). ML trees were then inferred in IQ-TREE with 1,000 ultrafast bootstrap replicates, and Maximum Likelihood bootstrap (MLB) values ≥ 70% are indicated at each node (Suppl. material 2: fig. S1).

### Genome sequencing, assembly, and gene prediction

The present study selected six *Pestalotiopsis* strains for whole genome sequencing (Table 1). These included three strains of *P. formosana* recovered from dead plant material, symptomatic tissue, and asymptomatic tissue, respectively, and two strains of *P. neolitseae* recovered from symptomatic tissue, enabling comparison of isolates associated with contrasting contexts. Genomic DNA extraction was performed using a modified CTAB extraction method (Doyle 1987; Liu et al. 2021). This study used the Illumina NovaSeq (Illumina, San Diego, CA, USA) platform, the Oxford Nanopore PromethION (Oxford Nanopore Technologies, Oxford, UK) platform, or both, to obtain WGS data from selected *Pestalotiopsis* strains. The sequencing services and related procedures were performed by BIOTOOLS Co., Ltd (New Taipei City, Taiwan) and the Biodiversity and Climate Research Centre, Goethe University (Germany). A summary of the genome-sequencing platform for each strain including WGS data retrieved from GenBank Genome database is provided in Suppl. material 1: table SS2.

Strains with only Illumina reads available were assembled as follows: Trimmomatic v.0.39 (Bolger et al. 2014) was employed to eliminate low-quality Illumina raw reads, the most prevalent adapters (Nextera transposase adapter sequence), and the first 20 base pairs of reads with suboptimal CG content. Fastp v.0.23.4 (Chen et al. 2018) was used for further filtering out low-quality raw reads and other adapters. FastQC v.0.12.1 (Andrews 2010) was utilized for quality control on each generated dataset. The best k-mer value was determined by Kmergenie v.1.7051 (Chikhi and Medvedev 2014). Finally, the Illumina raw read data were assembled using SPAdes v.3.15.5 (Bankevich et al. 2012). The Nanopore raw reads were filtered and trimmed using Porechop v.0.2.4 (Wick et al. 2017) and Filtlong v.0.2.1 (https://github.com/rrwick/Filtlong). Nanoplot v.1.20.0 (de Coster and Rademakers 2023) was utilized for quality control on each generated dataset. Finally, the raw read data were assembled by Flye v.2.9.3 (Kolmogorov et al. 2019). The strains sequenced with both the Illumina and Nanopore platforms were first *de novo* assembled by Nanopore long-reads, and later, polished by Illumina short reads with Pilon v.1.24 (Walker et al. 2014) four times to obtain the final genome.

All the genomes were processed using seqkit v.2.8.2 (Shen et al. 2016) to sort the sequences from largest to smallest and to remove fragments smaller than 1000 bp. Repeats and low-complexity sequences in the genomes were soft-masked using RepeatMasker v.4.1.5 with slow mode (option -s) and RMBlast searching engine (Tarailo-Graovac and Chen 2009) implemented with RepeatModeler v.2.0.5. to generate the custom repeat library with an LTR structural discovery pipeline for each strain (Flynn et al. 2020). The annotation results from RepeatMasker were subsequently summarized to assess transposable element (TE) composition and abundance across strains (Suppl. material 1: table S8). Assembly statistics and quality assessment of the genome were estimated in QUAST v.5.2.0 (Gurevich et al. 2013). An *ab initio* genome annotation method, the BRAKER3 pipeline was used to predict the proteins in the selected genomes (Stanke et al. 2006; Stanke et al. 2008; Brůna et al. 2021; Gabriel et al. 2024). The masked genomes were used to predict the protein using BRAKER v.3.0.8. The completeness of the genome and predicted proteins were assessed using BUSCO v.5.8.3 (Benchmarking Universal Single-Copy Orthologs)(Manni et al. 2021) with Fungi (n = 1,122), Ascomycota (n = 2,826), and *Sordariomycetes* (n = 4,492) lineage datasets from OrthoDB v.12 (Kriventseva et al. 2019).

### Identification of orthologous proteins

The orthologous proteins of *Pestalotiopsis* strains used in this study were identified by OrthoFinder v.2.5.5 (Emms and Kelly 2019), and the results were further interpreted using UpSetR in the R program (Conway et al. 2017). To evaluate whether ortholog content varied among isolates assigned to different ecological categories, ortholog comparisons between the three *P. formosana* isolates and the two *P. neolitseae* isolates were examined in parallel with genus-wide comparisons.

### Secretome and effectors identification

SignalP v.6.0 (Teufel et al. 2022) with the slow-sequential model was used to predict proteins containing signal peptides, and only proteins with a likelihood probability greater than 0.5 were selected for further analysis. DeepTMHMM v.1.0 was used to identify the presence of transmembrane domains in the proteins (Hallgren et al. 2022). Proteins with signal peptides but without transmembrane domains were classified as secretome (Forrester et al. 2014). The protein sequences identified as secretome were then used as input for effector prediction using EffectorP v.3.0 in FUNGAL_MODE (Sperschneider and Dodds 2022).

### Functional annotation of predicted proteins

Carbohydrate-active enzymes (CAZymes) were predicted on the dbCAN3 web server (Zheng et al. 2023) with three search pipelines, and only CAZymes annotated by at least two of the following approaches were considered: HMMER: dbCAN (E-value < 1e-15, coverage > 0.35), DIAMOND: CAZy (E-value < 1e-102), and HMMER: dbCAN-sub (E-value < 1e-15, coverage > 0.35). The trophic lifestyles of *Pestalotiopsis* taxa were inferred according to the CAZymes gene content using the CAZyme-Assisted Training And Sorting of -trophy (CATAStrophy) prediction tool v.0.1.0, using the v.8 model (Hane et al. 2020), and the principal components of *Pestalotiopsis* proteomes were output along with the training data samples of CATAStrophy. The dbCAN dataset (Zhang et al. 2018) was scanned by HMMER (Eddy 2011) using the hmmscan function. Clusters of Orthologous Groups (COG) were classified using eggNOG-mapper-2.1.12 in Diamond mode (Cantalapiedra et al. 2021). Additional pathogenicity-related proteins were identified through the BLASTP search against the PHI-base v.5.0 (Pathogen-Host Interactions database; http://www.phi-base.org/) (Urban et al. 2022).

### Secondary metabolite gene clusters identification

The genome FASTA files and the GFF3 files of *Pestalotiopsis* strains were used as input for antiSMASH fungal version v.8.0 (https://fungismash.secondarymetabolites.org/#!/start) (Blin et al. 2023) with relaxed detection strictness.

### General carbon utilization based on the Biolog FF microplate

Biolog FF microplates (Biolog Inc., USA) were used to test the global phenotypes and carbon source utilization of selected *Pestalotiopsis* strains representing different observed ecological contexts. The Biolog FF microplate contained 95 wells with different carbon-containing substrates and one well with water as a control (Suppl. material 1: table SS3).

Selected fungal strains were first inoculated onto MEA (Malt Extract Agar, HIMEDIA) and incubated at 25 ± 2 °C in the dark for seven days. The cultures were then transferred to constant blue light conditions and incubated at 25 ± 2 °C for 14 days to promote sporulation. After incubation, conidia were harvested and suspended in an inoculating fluid containing 0.25% gellan gum (Sigma-Aldrich) and 0.03% Tween 40 (Sigma-Aldrich) per liter. The conidial suspension was adjusted to 2 × 10^5^ spores/mL. The spore suspensions were inoculated into the Biolog FF microplate at 100 μL per well and incubated at 25 ± 2 °C in the dark for 240 hours. Initial absorbance readings (T = 0) were taken 30 minutes after inoculation using an EMax Plus microplate reader equipped with Softmax Pro 3 software, measuring at wavelengths of 490 nm and 750 nm. Subsequent reading was taken every 24 hours using the same measurement parameters. Each strain was analysed with five replicates across two independent trials. Statistical analyses were performed using formulas described by the previous literature (Garland and Mills 1991; Klimek and Niklińska 2007; Pawlik et al. 2019; Chou et al. 2022).

### General carbon utilization based on the FUNG-GROWTH database

Because the observed ecological contexts are often associated with expectations of metabolic specialization, carbon-use data for *P. formosana* isolates recovered from contrasting observed contexts (endophytic, diseased plants, saprobic) were compared directly to examine whether nutritional physiology showed consistent differentiation. All the strains used in the Biolog FF microplate assay were grown on minimal media (MM) supplemented with different carbon sources: monosaccharides (25 mM), polysaccharides (1%), and crude substrates (3%). A total of 20 carbon sources were tested in this experiment, and their complete details are provided in Suppl. material 1: table SS4. The composition of the minimal media per liter is as follows: 6.0 g of NaNO3, 1.5 g of KH2PO4, 0.5 g of KCl, 0.5 g of MgSO4 · 7H2O, and 200 μL of trace elements solution. The trace elements solution contains per liter: 10 g of EDTA, 4.4 g of ZnSO4 · 7H2O, 1.01 g of MnCl2 · 4H2O, 0.32 g of CoCl2 · 6H2O, 0.315 g of CuSO4 · 5H2O, 0.22 g of (NH4)6Mo7O24 · 4H2O, 1.47 g of CaCl2 · 2H2O, and 1.0 g of FeSO4 · 7H2O. Each plate was prepared with 12 mL of this minimal media. The cultures were incubated in darkness at 25 ± 2 °C until the fastest-growing colony reached the edge of the plate. Colony diameters were then measured for all tested strains. To assess differences in metabolic intensity and vegetative growth among strains, one-way analysis of variance (ANOVA) followed by Tukey’s honestly significant difference (HSD) post-hoc test were performed using Python v3.11.15 with SciPy (Virtanen et al. 2020) and statsmodels (Seabold and Perktold 2010) packages in Python.

## Results

### Identification of *Pestalotiopsis* species used for genome sequencing

The results of the multi-locus phylogeny based on DNA sequence data from ITS, tub2, and tef1-α are presented in Suppl. material 2: fig. S1. As the primary objective of this study was to compare strains of *P. formosana* and *P. neolitseae* recovered from contrasting plant contexts, the multilocus phylogeny was also used to confirm the conspecific placement of all focal strains. *Pestalotiopsis* sp. NC0098 clustered with the ex-type strain of *P. camelliae-oleiferae* CSUFTCC 08, showing low genetic divergence. In addition, *Pestalotiopsis* sp. 9143b clustered in the clade containing the ex-type strain of *P. hainanensis* PSHI2004Endo166, also exhibiting minimal genetic distance. Thus, the present study tentatively identifies these two strains as *P. camelliae-oleiferae* NC0098 and *P. hainanensis* 9143b, respectively. Furthermore, a strain designated as *P. neglecta* YJ-3 and *Pestalotiopsis* sp. JCM9685 were found within the same clade as the ex-type strain of *P. clavata* MFLUCC 12-0268. However, neither of these strains is linked to any morphological data. In addition, DNA sequence data from the ex-type strain of *P. neglecta* are currently unavailable. Based on these observations and their phylogenetic placement, the present study tentatively considers strains YJ-3 and JCM9685 to represent *P. clavata*. Notably, none of the genomes generated in this study clustered with any *Pestalotiopsis* genomes available from NCBI. These genomes include *P. formosana* (NTUCC 17-009, NTUPPMCC 21-035, NTUPPMCC 22-221), *P.*neolitseae (NTUCC 17-011, NTUPPMCC 21-036), and *P. sonneratiae* (NTUPPMCC 21-044). The three *P. formosana* strains clustered tightly with minimal phylogenetic divergence despite having been recovered from different hosts and plant contexts. Similarly, the two *P.*neolitseae strains showed no meaningful genetic distance. These placements indicate that contrasting sampling contexts do not correspond to detectable phylogenetic or species-level distinctions.

### Genome sequencing and general features

A total of ten *Pestalotiopsis* genomes including six newly generated genomes in this study and four strains from NCBI GenBank were included in the analysis (Suppl. material 1: table SS2). Their general genomic features, such as the number of contigs, total genome length, GC content, largest contig size, N50, L50, sequencing coverage, predicted protein numbers, and genome completeness assessments (Table 2; Supplementary tables S5–7). The GC content of the *Pestalotiopsis* strains showed little variation, ranging from 51.68% to 52.09%. The genome sizes varied from 46.25 to 48.35 Mbp. In this study, the number of contigs in the *Pestalotiopsis* genome assemblies ranged from 7 to 155, generally correlating with the sequencing technology employed. A contig count as low as 7 indicates a chromosome-scale assembly. Strains sequenced exclusively using second-generation technologies (Illumina platform) tended to have higher contig numbers, whereas those sequenced with a combination of second- and third-generation platforms, or using third-generation methods alone, typically exhibited fewer contigs. However, two strains deviated from this general trend. For instance, *P. hainanensis* 9143b, originally obtained from GenBank and sequenced using Nanopore technology, had 104 contigs. According to QUAST results, assembly mismatches were not high, suggesting that the elevated contig count is unlikely due to assembly errors (Gurevich et al. 2013). Instead, it may reflect low DNA quality or other technical limitations. In contrast, *P. clavata* JCM 9685, which was sequenced solely using the Illumina platform, had only 13 contigs, indicating a highly contiguous genome. Nonetheless, the assembly showed a high mismatch rate (97.81 N’s per 100 kbp), implying that the continuity may be compromised by a large number of uncalled bases, possibly due to low-quality reads or assembly artifacts. Unfortunately, the available data were insufficient to fully explain these discrepancies. Genome completeness of all strains included in this study was assessed using BUSCO v.5.8.3. The results showed that all *Pestalotiopsis* genomes exhibited over 95% completeness at the Fungi, Ascomycota, and *Sordariomycetes* lineage levels (Suppl. material 1: table S6). The predicted number of protein-coding genes across the strains ranged from 14,003 to 14,936.

**Table 2.** Genomic features of *Pestalotiopsis* strains analysed in this study.

Taxon	Strain	Contigs	Total length (mbp)	Genome BUSCO (%)	GC (%)	Predicted proteins	Protein BUSCO (%)
*P. camelliae-oleiferae*	NC0098	24	46.40	98.8	52.01	14,550	98.9
*P. clavata*	JCM9685	13	48.23	99.0	51.68	14,491	99.3
	YJ-3	8	48.35	99.1	51.73	14,540	99.3
***P. formosana***	**NTUCC 17-009**	**87**	**46.53**	**99.0**	**52.02**	**14,619**	**99.2**
	**NTUPPMCC 21-035**	**9**	**46.65**	**99.0**	**52.02**	**14,695**	**99.3**
	**NTUPPMCC 22-221**	**11**	**46.59**	**99.0**	**52.03**	**14,663**	**99.2**
*P. hainanensis*	9143b	104	46.25	96.3	52.03	14,102	96.5
***P. neolitseae***	**NTUCC 17-011**	**7**	**46.84**	**99.0**	**52.06**	**14,936**	**99.2**
	**NTUPPMCC 21-036**	**15**	**46.80**	**98.9**	**52.09**	**14,577**	**99.3**
***P. sonneratiae***	**NTUPPMCC 21-044**	**155**	**46.42**	**99.0**	**52.01**	**14,575**	**99.3**
*Ps. fici*	W106-1	39	51.91	99.1	48.73	15,708	99.5

Notes: Assembly statistics and GC content were estimated using QUAST v.5.2.0. Genome completeness and predicted protein-coding gene content were assessed with BUSCO v.5.8.3 in both genome and protein modes, using the *Sordariomycetes* lineage dataset (n = 4,492) from OrthoDB v.12. Strains in bold indicate newly sequenced whole-genome assemblies generated in this study.

Notably, the three *P. formosana* strains exhibited nearly identical genome sizes (46.53–46.65 Mbp), GC content (~52.02–52.03%), and predicted proteins (14,619–14,695), despite having been recovered from dead plant material, symptomatic tissue, and asymptomatic tissue. Likewise, the two *P. neolitseae* strains (NTUCC 17-011 and NTUPPMCC 21-036), both recovered from symptomatic tissues, were almost indistinguishable in general genome features. These results indicate that neither genome assembly quality nor core genomic architecture shows a clear association with the isolation contexts.

### Ortholog group analysis

**Figure 1.**
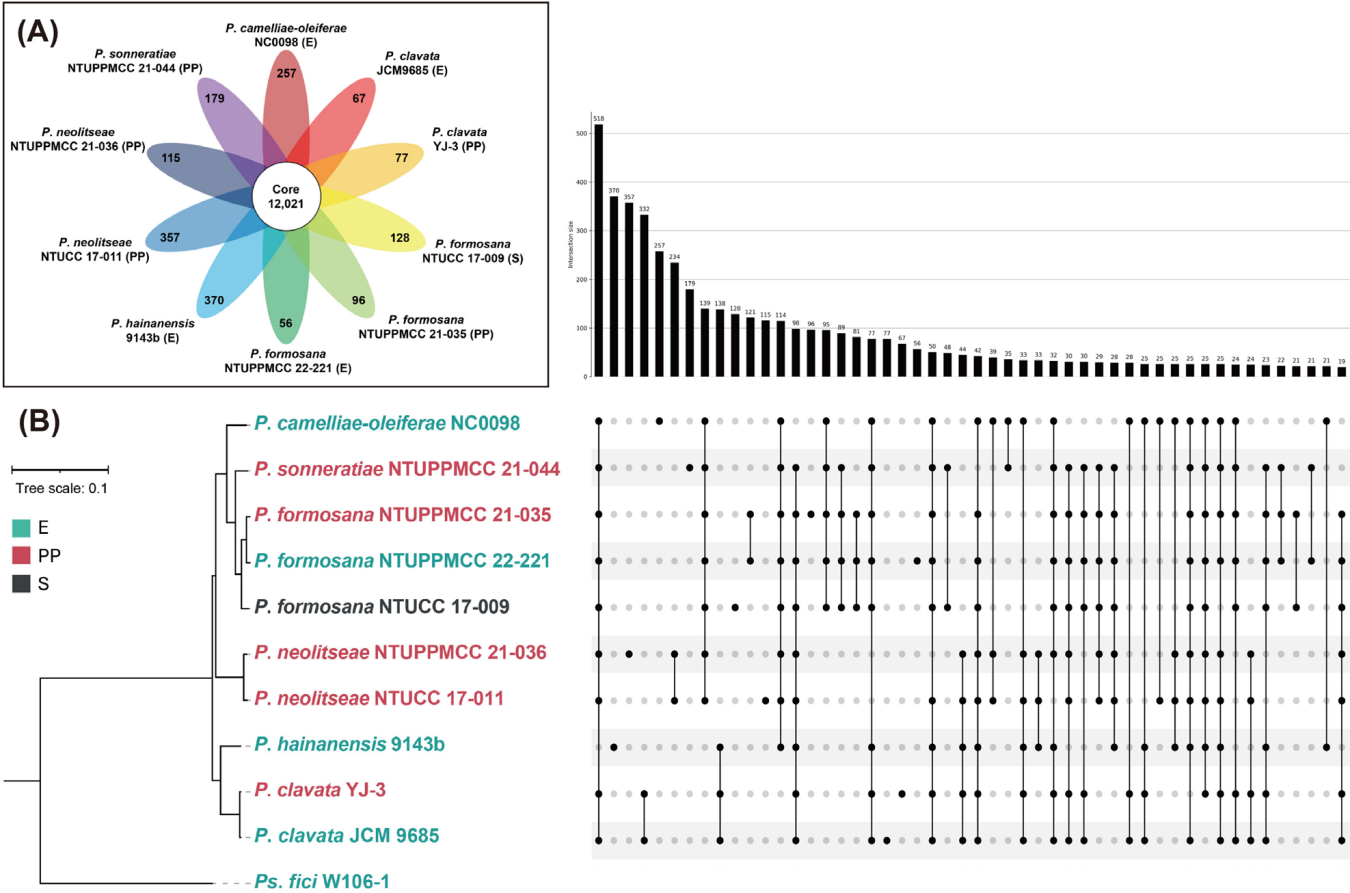
Flower plot and UpSet diagram showing shared orthologous proteins among *Pestalotiopsis* strains analyzed in this study. (A) A total of 12,021 core orthologous proteins were shared among the ten *Pestalotiopsis* strains, and the number of strain-specific orthologous proteins for each species is indicated in the ellipse (E, isolate recovered from asymptomatic tissue; PP, isolate recovered from symptomatic tissue; S, isolate collected from dead or decaying material). (B) The phylogenetic tree was inferred with OrthoFinder based on multiple sequence alignments and constructed using FastTree. *Pseudopestalotiopsis fici* W106-1 was used as outgroup. The scale bar indicates the number of estimated substitutions per site. The UpSet plot to the right of the phylogenetic tree illustrates the intersections of orthologous proteins. Orthologous proteins shared across all *Pestalotiopsis* strains and strain-specific orthologous proteins are excluded; only the 50 largest orthologous groups are displayed.

In total, 12,021 core orthologous genes were identified as shared among the ten *Pestalotiopsis* strains analysed in this study (Fig. 1). These core orthologous genes are conserved across all strains, indicating a high degree of genomic conservation across the taxa examined in the present study. When *P. formosana* and *P. neolitseae* strains were compared exclusively, all analysed isolates shared essentially the same orthologous gene content, with no orthologs consistently associated with the observed ecological contexts. Importantly, the three *P. formosana* strains did not differ in orthologous groups despite having been recovered from dead material, symptomatic tissue, and asymptomatic tissue. Similarly, the two *P.*neolitseae strains showed full overlap with no orthologs unique to the symptomatic-tissue isolates.

### Secretome and effectome identification

Predicted secretome proteins ranged from 1,706 to 1,854 (~12.06–12.64% of total proteins), and predicted effectome proteins from 425 to 447 (~2.92–3.08% of total proteins or ~23.52–25.01% of the secretome) across the ten *Pestalotiopsis* genomes analyzed in this study. Within *P. formosana*, isolates recovered from dead material (NTUCC 17-009), symptomatic tissue (NTUPPMCC 21-035), and asymptomatic tissue (NTUPPMCC 22-221) showed almost identical secretome counts (1,841–1,854). Effectome content was likewise very similar (433–443 predicted effectors), demonstrating no consistent increase or decrease corresponding to the observed ecological contexts. Similarly, the two *P. neolitseae* strains recovered from symptomatic tissue showed similar secretome and effectome sizes. Overall, these comparisons suggest that secretome and effectome contents in *Pestalotiopsis* are largely conserved across the sampled plant contexts. These findings show that the general abundance of secreted proteins and effectors does not explain the different ecological associations reported for these species.

### CAZymes analysis

Across all *Pestalotiopsis* strains examined, the total CAZyme count ranged from 859 to 914 genes (Suppl. material 1: table S9). Glycoside hydrolases (GH) were the most abundant group (~45.1–46.0%), followed by auxiliary activities (AA; ~27.0–28.3%). Across the observed ecological contexts, the overall distribution of major families (GH, GT, CE, CBM, and PL) was highly similar (Suppl. material 2: fig. S2). A core set of enzyme families, specifically AA7 (75–83 genes), AA3 (63–69 genes), and GH3 (35–38 genes), was consistently abundant across all genomes (Suppl. material 1: table S10). Even at the subfamily level (e.g., AA1, AA3, AA7), we found no significant differences corresponding to the observed ecological contexts. Furthermore, substrate-specific analysis confirmed that all strains possess extensive genes associated with plant polysaccharides (cellulose, hemicellulose, and pectin), consistent with the genus’s common association with plants (Suppl. material 1: table S11).

**Figure 2.**
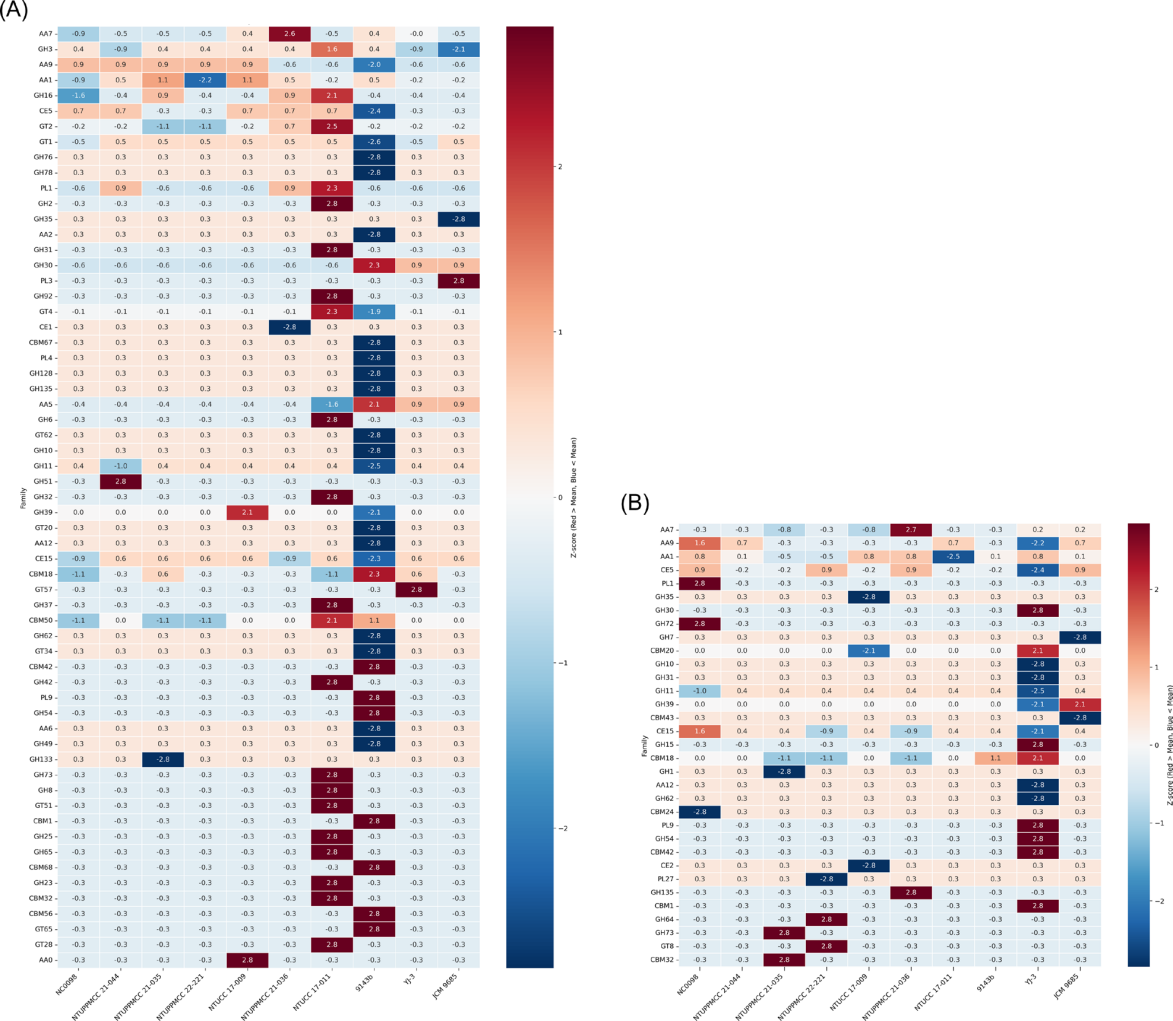
Z-score normalized heatmaps of (A) total genomic and (B) secreted CAZymes profiles of *Pestalotiopsis* strains. The colour scale represents the number of standard deviations from the genus mean, where red denotes expansion and blue denotes contraction. Families with invariant gene counts across all strains were omitted to highlight divergent profiles.

While grouping by the observed ecological contexts yielded no clear distinctions, Z-score analysis highlighted statistically significant variation at the strain level (Fig. 2). However, it is essential to note that these high Z-scores often reflected subtle numerical differences rather than extensive genomic alterations. For instance, the pathogenic *P. neolitseae* strain NTUCC 17-011 exhibited a statistically significant expansion in families such as GH31 (9 genes) and GH25 (1 gene), yet these counts exceeded the genus average by a narrow margin (Suppl. material 1: table S10) (mean ≈ 8.1 and 0.1, respectively). Similarly, the distinct profile of the *P. hainanensis* (9143b), characterized by reductions in certain families or enrichments in binding modules (e.g., CBM1, 2 genes), was driven by small absolute deviations from the mean (mean ≈ 0.1). These findings suggest that strain-specific divergence in *Pestalotiopsis* involves fine-scale copy number variations, often differing by only a single gene rather than fundamental functional shifts.

Analysis of the predicted secretome—representing about 60% of the total CAZymes count (range from 519 to 545 genes, Suppl. material 1: table S12)—further highlighted the functional flexibility within the genus. Although the total number of secreted CAZymes was similar across strains, Z-score analysis identified statistically significant enrichments in specific families, though these often involved small integer changes in gene copy number (Suppl. material 1: table S13). Notably, *P. clavata* YJ-3 exhibited a unique secretion profile, being the only strain to encode secreted CBM1 (2 genes) in this dataset. It also showed significant Z-score expansions in families such as GH54 (2 genes) and GH30 (9 genes), although these counts exceeded the genus only slightly (averages of 1.1 and 7.2, respectively). In contrast, *P. camelliae-oleiferae* NC0098 showed statistically high Z-scores for PL1 (14 genes) and GH72 (7 genes), despite these counts being very close to the genus average (means of 13.1 and 6.1, respectively) (Suppl. material 1: tables S13, S14). These profiles suggest that *Pestalotiopsis* species possess a flexible enzymatic toolkit, where subtle, strain-specific variations in secreted enzymes rather than large-scale genomic restructuring, may support different ecological strategies.

**Figure 3.**
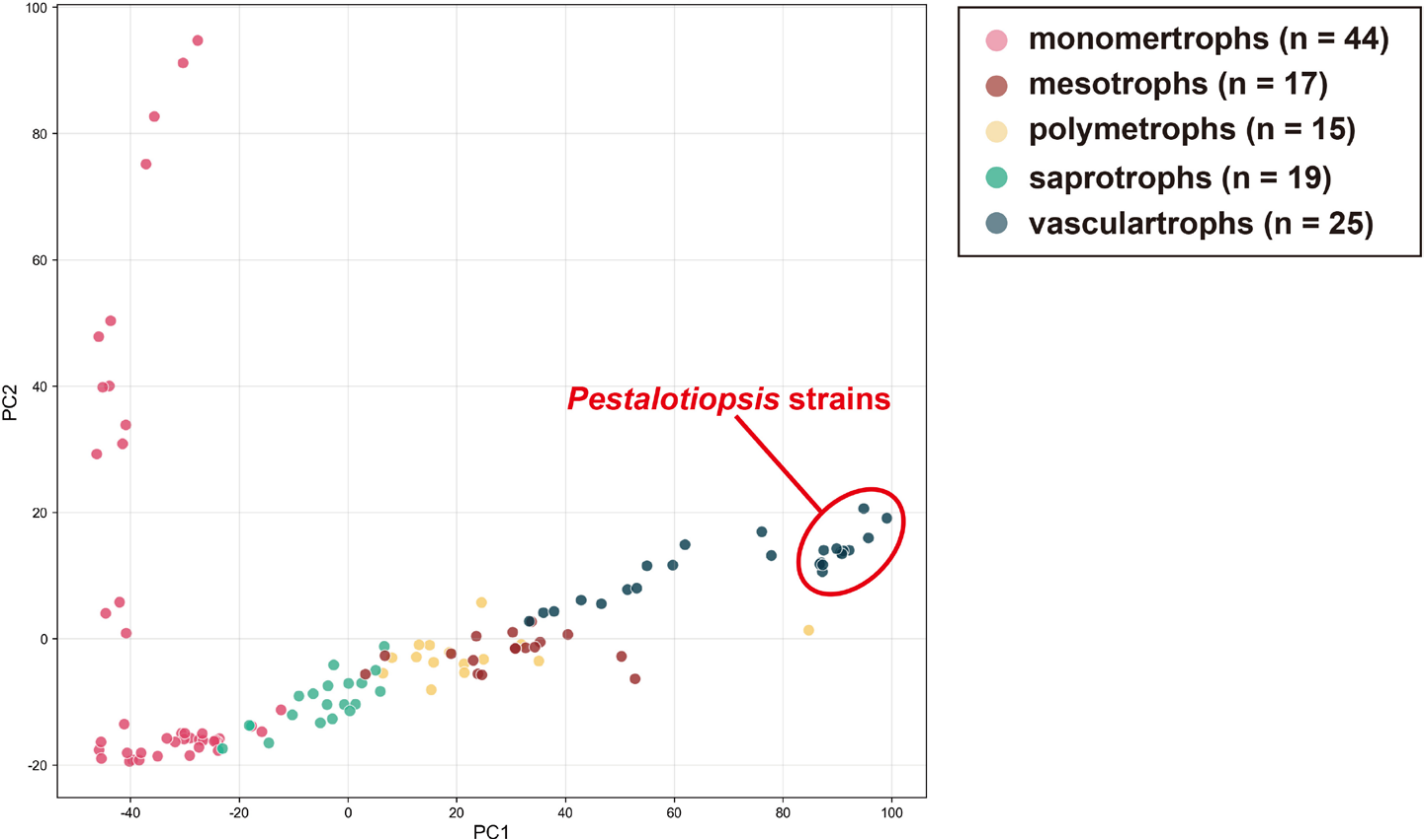
PCA plot of CATAStrophy lifestyle predictions, with *Pestalotiopsis* strains outlined in a red circle.

Consistent with the similarity observed in genomic CAZymes, trophic prediction using CATAStrophy placed all analysed strains in the vasculartroph category (Table 3, Suppl. material 1: table S15, Hane et al. 2020; Fig. 3). This prediction groups them with fungi having broadly similar CAZyme-content profiles, including known vascular pathogens such as *Colletotrichum gloeosporioides* and *Fusarium oxysporum*. However, this result should be interpreted as a CAZyme-content-based trophic prediction rather than as direct evidence of natural ecological behavior. In this context, the shared prediction is consistent with the broader genomic similarity observed among the sampled strains.

**Table 3.** Prediction of trophic lifestyles of *Pestalotiopsis* genomes based on CAZyme content using CATAStrophy v.0.1.0 (model v.8).

Taxon	Strain	CATAStrophy prediction				
		ME	MO	P	S	V
*Pestalotiopsis camelliae-oleiferae*	NC0098	0.73	0.00	0.72	0.23	1.00
*Pestalotiopsis clavata*	JCM9685	0.73	0.00	0.71	0.23	1.00
	YJ-3	0.74	0.00	0.72	0.24	1.00
***Pestalotiopsis formosana***	**NTUCC 17-009**	**0.73**	**0.00**	**0.71**	**0.23**	**1.00**
	**NTUPPMCC 21-035**	**0.73**	**0.00**	**0.71**	**0.23**	**1.00**
	**NTUPPMCC 22-221**	**0.73**	**0.00**	**0.71**	**0.23**	**1.00**
*Pestalotiopsis hainanensis*	9143b	0.73	0.00	0.71	0.23	1.00
***Pestalotiopsis neolitseae***	**NTUCC 17-011**	**0.73**	**0.00**	**0.72**	**0.23**	**1.00**
	**NTUPPMCC 21-036**	**0.72**	**0.00**	**0.71**	**0.23**	**1.00**
***Pestalotiopsis sonneratiae***	**NTUPPMCC 21-044**	**0.74**	**0.00**	**0.72**	**0.24**	**1.00**

Notes: All *Pestalotiopsis* genomes available in the NCBI database, as well as the complete genomes of the strains analysed in this study, were identified as vasculartrophic fungi (V, vascular pathogens), regardless of their original lifestyle designations. Strains shown in bold indicate newly sequenced whole-genome assemblies generated in this study. Trophic classes are abbreviated as follows: MO, monomertrophs; ME, mesotrophs; P, polymetrophs; S, saprotrophs; V, vasculartrophs. Relative centroid distances (RCDs) of each strain. The closest centroid was assigned an RCD value of 1, the furthest a value of 0, with other centroid distances represented as relative proportions. Species were classified into a major trophic class when RCD = 1, and into sub-classes when RCD ≥ 0.8.

### Functional analysis

#### Clusters of orthologous genes

Proteins assigned to COG functional categories (Galperin et al. 2021) were highly conserved across the ten *Pestalotiopsis* genomes analysed in this study (Suppl. material 1: table S16). Within *P. formosana*, isolates recovered from dead material (NTUCC 17-009), symptomatic tissue (NTUPPMCC 21-035), and asymptomatic tissue (NTUPPMCC 22-221) showed almost identical counts in the Metabolism category (4,257–4,276 proteins). In addition, content in Cellular processes and signalling was likewise very similar (1,906–1,928 proteins), demonstrating no consistent increase or decrease corresponding to the observed ecological contexts. Similarly, the two *P. neolitseae* strains showed similar functional profiles. Overall, core functional categories in *Pestalotiopsis* appeared largely conserved across the sampled plant contexts. These findings show that general genomic functional capacity does not explain the different ecological associations reported for these species.

#### PHI-base

To determine whether the observed ecological contexts were reflected in specific virulence-associated gene sets, the present study annotated the genomes and secretomes of *P. formosana* and *P. neolitseae* using the PHI-base database (Suppl. material 1: tables S17, S18). In the genome-wide analysis of *P. formosana*, the abundance of candidate pathogenicity-related genes was remarkably consistent across isolates. For instance, the isolate from dead material (NTUCC 17-009) harboured 5,290 PHI-base homologs, a number similar to the isolates from symptomatic tissue (NTUPPMCC 21-035; 5,286 homologs) and asymptomatic tissue (NTUPPMCC 22-221; 5,251 homologs). This uniformity was even more noticeable in the secreted fraction of the proteome, where the total count of potential virulence factors in *P. formosana* ranged narrowly from 672 to 678. Similarly, the two *P. neolitseae* strains exhibited matching profiles without major differences. The absence of distinct grouping among these isolates indicates that the broad genomic machinery associated with pathogenicity-related functions is present across the sampled strains, irrespective of their observed ecological contexts.

### Secondary metabolite gene clusters (SMGCs)

Genome mining with antiSMASH revealed that the potential for secondary metabolites is robust and consistently present across the sampled *Pestalotiopsis* isolates (Suppl. material 1: table S19). The composition across different *Pestalotiopsis* strains was similar. NRPS genes were the most abundant (29–34), followed by PKS genes (21–33), terpene genes (20–27), and indole genes (4–5). RiPP genes (1–2) and others were the least abundant. Within *P. formosana*, the overall count of secondary metabolite gene clusters (SMGCs) was tightly clustered, with the isolate from dead material (NTUCC 17-009) harboring 91 clusters, a figure nearly identical to the isolates from symptomatic tissue (NTUPPMCC 21-035; 88 clusters) and asymptomatic tissue (NTUPPMCC 22-221; 87 clusters). A detailed breakdown showed that while non-ribosomal peptide synthetases (NRPS) were perfectly conserved at 32 genes across all three isolates, the dead-material isolate possessed a slight numerical advantage in polyketide synthases (PKS, 29 genes) compared to the other two isolates (26 genes). In the case of *P. neolitseae*, the two strains exhibited moderate variation, particularly in NRPS content (29 vs. 33 genes), yet their total SMGC counts remained comparable (88 vs. 91). Across all analysed genomes, NRPS and PKS clusters were the dominant classes, followed by terpenes and indoles. Comparisons revealed that no specific SMGC category was clearly enriched or reduced in isolates from symptomatic tissue relative to the other sampled plant contexts.

### Analysis of transposable elements

RepeatMasker analysis revealed that transposable elements (TEs) in *Pestalotiopsis* strains contributed to a low percentage of the genome assemblies, ranging from 0.68% to 3.40% (Suppl. material 1: table S8). Among the identified TE classes, LTR retrotransposons were the main components, accounting for the vast majority of TE content, with the Gypsy superfamily representing the major fraction (data not shown). However, LINEs and DNA transposons were present only at low levels or nearly absent. A significant fraction of repeats, however, remained unclassified and contributed consistently across all genomes. Our genomic survey via RepeatMasker demonstrated that the repeat landscape in *P. formosana* and *P. neolitseae* is limited and does not correlate clearly with the observed ecological contexts. Total interspersed repeats occupied a minimal fraction of the genome assemblies. In *P. formosana*, the isolate from dead material (NTUCC 17-009) contained 0.89% repetitive content, a figure that was similar to the isolates from symptomatic tissue (NTUPPMCC 21-035; 0.77%) and asymptomatic tissue (NTUPPMCC 22-221; 0.68%). Similarly, the two *P. neolitseae* strains exhibited comparable TE loads, ranging narrowly from 1.34% to 1.40%. Regarding composition, LTR retroelements were the most prevalent class identified, whereas DNA transposons and LINEs were very low or absent. Although some variation in total TE content existed across the wider genus, the data suggest there is no clear increase of transposable elements associated with the observed ecological contexts.

### Nutrient utilization test

#### Biolog FF microplate

Metabolic profiling via the Biolog FF system revealed that all analysed *Pestalotiopsis* strains are nutritional generalists capable of utilizing a broad range of substrates, although clear quantitative differences in metabolic intensity were observed. In terms of substrate breadth, all strains showed high substrate richness (RS), ranging from 82 to 91, confirming a shared capability to degrade diverse carbon sources. However, the intensity of this metabolism (average well colour development, AWCD) was notably higher in the *P. formosana* isolate recovered from dead material (NTUCC 17-009), which reached an AWCD of 0.694, compared with the isolates recovered from symptomatic tissue (NTUPPMCC 21-035; 0.558) and asymptomatic tissue (NTUPPMCC 22-221; 0.532) (Table 4; Suppl. material 2: fig. S3). This trend of elevated metabolic activity in the dead-material isolate was consistent across multiple carbon guilds, including carbohydrates, amino acids, and polymers. Regarding activity over time, most strains exhibited a rapid onset of metabolism within the first 72 hours. An exception was observed in *P. neolitseae* NTUCC 17-011, which displayed an initial lag phase before accelerating between 48 and 96 hours to eventually converge with the metabolic profiles of the other strains. By the 168-hour endpoint, despite the differences in intensity, each strain successfully metabolized ~90% of the 95 tested substrates (Suppl. material 1: table S20; Suppl. material 2: fig. S3A), with only four substrates consistently unutilized across the group. These findings indicate that while the fundamental metabolic potential is conserved across the sampled strains, the efficiency or regulation of nutrient uptake may vary *in vitro*.

**Table 4.** Overall results of Biolog FF microplate assay.

Taxon	Strain	Observed ecological context	RS	AWTD	AWCD
*Pestalotiopsis formosana*	NTUCC 17-009	S	91	0.865	0.694
	NTUPPMCC 21-035	PP	87	1.036	0.558
	NTUPPMCC 22-221	E	85	0.890	0.532
*Pestalotiopsis neolitseae*	NTUCC 17-011	PP	82	0.980	0.571
	NTUPPMCC 21-036	PP	82	0.898	0.557
*Pestalotiopsis sonneratiae*	NTUPPMCC 21-044	PP	85	0.935	0.581

Notes: RS, substrate richness; AWCD, average well colour development; AWTD, average well turbidity development; S, isolate collected from dead or decaying material; PP, isolate recovered from symptomatic tissue; E, isolate recovered from asymptomatic tissue

### Carbon source media

To assess metabolic versatility on solid substrates, we cultured the *Pestalotiopsis* strains on 20 distinct media formulations (19 supplemented with specific carbon sources and one unsupplemented control) to evaluate growth patterns (Suppl. material 1: table S21; Suppl. material 2: fig. S4). Consistent with the Biolog results, all strains thrived on crude plant-derived substrates, with maximal colony expansion observed on rice bran, wheat bran, and soybean hulls. Regarding pure substrates, while simple sugars (glucose and sucrose) supported strong growth, polysaccharide utilization varied significantly. For comparison, growth on minimal medium without added carbon resulted in sparse mycelia with diameters of 42.8–57.6 mm. Interestingly, relative to this minimal medium baseline, vegetative growth was notably inhibited on citrus pulp (7.0–13.9 mm) and moderately reduced on lignin (25.2–31.1 mm) and citrus pectin (37.6–46.0 mm).

Unlike the broad genomic similarity, phenotypic growth assays revealed statistically significant variations in vegetative vigour that were strain-specific rather than clearly associated with the observed ecological context. For instance, within *P. formosana*, NTUPPMCC 21-035 exhibited robust growth on cellulose and rice bran compared with NTUPPMCC 22-221 and NTUCC 17-009. Yet, this enhanced vigour was not a universal property of isolates recovered from symptomatic tissues; the two *P. neolitseae* isolates also differed markedly from one another, with NTUPPMCC 21-036 consistently outgrowing NTUCC 17-011 across nearly all carbon sources. These results confirm that while all tested *Pestalotiopsis* strains possess the fundamental metabolic machinery to degrade diverse plant polymers, their vegetative growth rates are profoundly influenced by strain-specific physiological factors.

When comparing these results to the Biolog FF microplate assays for common substrates (glucose, sucrose), neither OD_750_ (hyphal density) nor CRV (metabolic capability) correlated consistently with colony diameters on agar media (Suppl. material 1: table S22). This discrepancy suggests that microplate-based measures of growth and metabolic activity may not directly translate to colony expansion on solid substrates.

## Discussion

The comparative genomic and metabolic analyses conducted in this study show that *Pestalotiopsis formosana* and *P. neolitseae* share a high degree of genomic and functional conservation despite having been recovered from symptomatic, asymptomatic, and dead plant tissues. Within the present sampling framework, we did not detect obvious genome-scale signatures consistently associated with the observed ecological contexts. This finding directly addresses the longstanding uncertainty surrounding the true ecological identity of *Pestalotiopsis* species (Maharachchikumbura et al. 2014; Ariyawansa and Hyde 2018). Our results show that lifestyle labels assigned based on field observations or isolation source do not correspond to underlying genomic specialization. Instead, lifestyle expression in these taxa appears to be strongly context dependent, representing flexible interaction outcomes that differ according to host species, host condition, and environmental factors.

As the present sampling is limited and not fully balanced across the observed ecological contexts, the absence of detectable genomic differentiation should be further explored using larger datasets. However, the interpretation of the results of the current study is consistent with a large-scale comparative study on genomes in *Sordariomycetes*. Chen et al. (2023) showed that fungal lifestyles can be predicted to some extent from large-scale genomic datasets, particularly using functional protein repertoires, but also emphasized that predictive resolution depends strongly on label accuracy, sampling balance, and the degree of ecological specialisation. Their results further indicated that basic genomic features reflect phylogeny more strongly than lifestyle, which aligns with our observation that the analysed *Pestalotiopsis* isolates show no genome-scale differentiation despite contrasting observed ecological contexts. It thus seems likely that in the *Pestalotiopsis* species analysed, lifestyle expression resulting in the observed ecological categories is likely context dependent, representing flexible interaction outcomes that differ according to host species, host condition, and environmental factors.

Across all genomic features examined, including genome size, core orthologous content, CAZyme repertoires, secretomes, effectomes, secondary metabolite gene clusters, and transposable elements, *P. formosana* and *P. neolitseae* strains exhibited a remarkably conserved genomic architecture. Although fine-scale statistical analysis revealed subtle strain-specific expansions such as the enrichment of glycoside hydrolases in *P. neolitseae* NTUCC 17-011—the overall profiles remained broadly similar. Remarkably, *P. formosana* NTUCC 17-009, NTUPPMCC 21-035, and NTUPPMCC 22-221 showed virtually identical genomic signatures despite having been recovered from dead grass, diseased *Podocarpus*, and healthy *Podocarpus* leaves, respectively. Likewise, the two *P. neolitseae* strains shared extensive overlap in all examined metrics despite distinct host associations and observed ecological contexts. These results are consistent with the interpretation that these taxa possess a broadly shared genomic toolkit, while any ecological differences may depend more on regulation, physiology, host status, or environment than on major differences in gene content. Comparable observations have been made in other fungal groups where genomic content does not cleanly reflect ecological assignment, including fusarioid fungi and *Phyllosticta citrichinaensis* (Hill et al. 2022; Buijs et al. 2022; Ulaszewski et al. 2025).

CAZyme profiles are often informative in broad comparisons of plant-associated fungal trophic strategies (Zhao et al. 2014; Wang et al. 2022). In *Pestalotiopsis*, however, CAZyme repertoires were uniformly large and highly conserved among all analysed strains. While individual strains may exhibit targeted reinforcements such as the CBM enrichment in *P. clavata* YJ-3—no global reduction or expansion trend corresponded to the observed ecological contexts. Importantly, CATAStrophy placed all analysed strains in the vasculartroph category. This shared prediction should be interpreted cautiously, as it is inferred from CAZyme content and does not directly demonstrate natural ecological behaviour. Nevertheless, it is consistent with the broader result that the sampled strains occupy similar genomic trophic prediction space.

Secreted proteins, effectors, and secondary metabolites are often implicated in host specificity and pathogenicity (Hacquard et al. 2016; Stauber et al. 2020). In contrast, the *Pestalotiopsis* strains in our study showed no meaningful differences in these gene categories across the sampled plant contexts. The *P. formosana* isolate recovered from asymptomatic tissue possessed essentially the same secretome and effectome as the isolate recovered from symptomatic tissue, and no context-associated secondary metabolite clusters were detected. These results indicate that neither virulence-related gene content nor secondary metabolism alone explains the divergent ecological associations reported for these species. Because these candidate effectors were identified strictly through *in silico* structural predictions, their specific roles in host manipulation remain to be functionally validated. Given that the overall size of the effectome is conserved across the analysed isolates, any differences in ecological expression may depend more on context-dependent gene regulation than on major differences in predicted effector content. Future research, particularly regarding *in planta* transcriptomic profiling (RNA-seq) during active colonization, will be essential to decipher these dynamics. Such expression profiles could reveal whether specific subsets of this shared effector reservoir are differentially activated in response to host immune triggers or environmental shifts, thereby elucidating the mechanisms underlying their ecological plasticity.

Many rapidly evolving pathogens rely on TE-rich genome regions to diversify effector genes and accelerate adaptation (Raffaele and Kamoun 2012). Our TE analyses showed that *Pestalotiopsis* genomes are uniformly TE-poor (<5%), similar to other *Sordariomycetes* lacking accelerated genome compartments. The absence of TE-mediated “two-speed” genome architecture suggests that any ecological differentiation in the sampled isolates is unlikely to be driven by large-scale repeat-associated genome restructuring. Instead, ecological versatility appears to be encoded within a stable genomic framework (Wacker et al. 2023).

Carbon-source utilization, both in Biolog FF microplates and crude-substrate media, confirmed that the analysed strains share a broad, generalist metabolic potential. All *Pestalotiopsis* isolates metabolized similar numbers of substrates, reinforcing the genomic observation of a conserved CAZyme repertoire. However, phenotypic assays revealed quantitative variations in metabolic efficiency that were uncoupled from genome content. For example, the *P. formosana* isolate recovered from dead material (NTUCC 17-009) displayed higher metabolic intensity in microplate assays than the isolates recovered from symptomatic or asymptomatic tissues, despite possessing a nearly identical CAZyme repertoire. Likewise, growth vigour on solid media varied substantially among strains, including between the two *P. neolitseae* isolates. These findings support the view that a versatile metabolic toolkit is shared across the sampled strains, whereas *in vitro* expression of that potential is modulated by strain-specific physiological or regulatory differences.

Our findings are therefore consistent with a model of overlapping ecological versatility in *P. formosana* and *P. neolitseae*, in which behaviour may be shaped by multiple interacting factors, including fungal genotype, host identity and physiological state, environmental variables, and surrounding biotic context. It is important to note that in this conjunction that isolates recovered from symptomatic tissue are not automatically confirmed as primary pathogens, isolates from asymptomatic tissue may include latent or opportunistic pathogens, and isolates from dead material may include secondary colonizers rather than obligate saprobes (Blumenstein et al. 2021; Prechsl et al. 2023; Slippers et al. 2024). Thus, observed ecological contexts should only be viewed as a snapshot of the life history of an organism, rather than fixed categories.

Overall, the present study suggests that rather than fixing ecological identity of each isolate based on field observation, a more cautious interpretation of observed ecological contexts is necessary, because fungi that share a conserved genomic toolkit may express different ecological behaviours depending on context. Future investigations integrating transcriptomics, metabolomics, in planta assays, and environmental simulations will be essential for identifying the molecular and ecological mechanisms governing these lifestyles.

## Additional information

### Artificial Intelligence (AI) use

The authors accept full responsibility for the content of the manuscript, including the disclosure of any use of AI.

No AI tools were used in the preparation of this manuscript.

### Adherence to national and international regulations

All the fungal strains used in this study have been legally obtained, respecting the Convention on Biological Diversity (Rio Convention).

### Author ORCIDs

S.-Y. Hsu https://orcid.org/0000-0001-6227-0936

B. Mishra https://orcid.org/0009-0008-4925-0904

H.-Y. Huang https://orcid.org/0009-0009-2618-6257

Y.-C. Lin https://orcid.org/0000-0002-2319-0869

Y.-C. Xu https://orcid.org/0000-0002-3885-498X

S. Ploch https://orcid.org/0000-0002-7129-4523

C.-Y. Hsu https://orcid.org/0009-0009-7091-0695

J.-P. Wang https://orcid.org/0009-0008-6697-2167

A.M. Elgorban https://orcid.org/0000-0003-3664-7853

K.M. Alarjani https://orcid.org/0000-0002-3762-1498

M. Thines https://orcid.org/0000-0001-7740-6875

H.A. Ariyawansa https://orcid.org/0000-0001-8526-7721

### Data availability

All of the data that support the findings of this study are available in the main text or Supplementary materials. The Pestalotiopsis genome data generated in this study have been submitted to the NCBI BioProject database (https://www.ncbi.nlm.nih.gov/bioproject/) under accession numbers JBOZWL000000000 (NTUCC 17-009), JBOZWK000000000 (NTUPPMCC 21-035), JBOZWJ000000000 (NTUPPMCC 22-221), JBOZWE000000000 (NTUCC 17-011), JBOZWI000000000 (NTUPPMCC 21-036), and JBOZWH000000000 (NTUPPMCC 21-044).

## Supplementary materials

Supplementary material 1 Additional tables Data type xlsx https://binary.pensoft.net/file/1661174This dataset is made available under the Open Database License (http://opendatacommons.org/licenses/odbl/1.0/). The Open Database License (ODbL) is a license agreement intended to allow users to freely share, modify, and use this Dataset while maintaining this same freedom for others, provided that the original source and author(s) are credited. Sheng-Yu Hsu, Bagdevi Mishra, Hsin-Yao Huang, Yu-Chen Lin, Yuan-Cheng Xu, Sebastian Ploch, Chia-Ying Hsu, Jau-Ping Wang, Abdallah M. Elgorban, Khaloud M. Alarjani, Marco Thines, Hiran A. Ariyawansa

Supplementary material 2 Additional figures Data type docx https://binary.pensoft.net/file/1661175This dataset is made available under the Open Database License (http://opendatacommons.org/licenses/odbl/1.0/). The Open Database License (ODbL) is a license agreement intended to allow users to freely share, modify, and use this Dataset while maintaining this same freedom for others, provided that the original source and author(s) are credited. Sheng-Yu Hsu, Bagdevi Mishra, Hsin-Yao Huang, Yu-Chen Lin, Yuan-Cheng Xu, Sebastian Ploch, Chia-Ying Hsu, Jau-Ping Wang, Abdallah M. Elgorban, Khaloud M. Alarjani, Marco Thines, Hiran A. Ariyawansa
