# Supplementary material for: Genomic and metabolic uniformity across diverse observed ecological contexts suggest that interactions of Pestalotiopsis formosana and P. neolitseae are context-dependent
Source: IMA Fungus. 2026 May 28;17:e192779. doi: 10.3897/imafungus.17.192779 (PMC13237565; doi:10.3897/imafungus.17.192779)
Supplement: Supplementary material 2 — Additional figures [file imafungus-17-e192779-s002.docx]

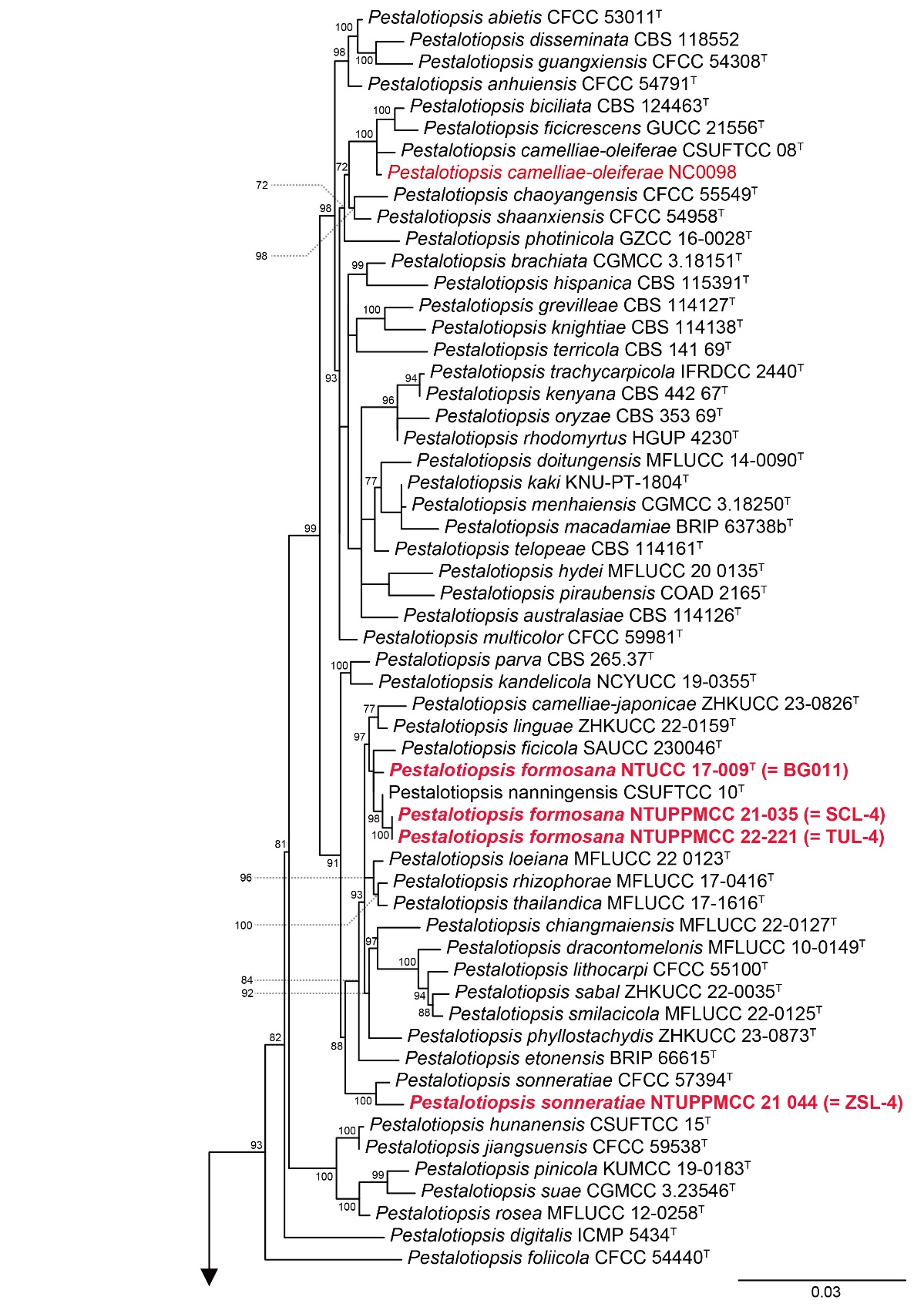


**Supplementary Figure S1.** Maximum likelihood phylogenetic tree of *Pestalotiopsis* obtained from the concatenated DNA sequence data of ITS, *tub2*, and *tef1-α* implemented in IQ-TREE. ML bootstrap values (MLB) ≥ 70% are given at the nodes. The scale-bar shows the number of estimated substitutions per site. *Neopestalotiopsis* *protearum* (CBS 114178) was used as an outgroup in this study. The strains in red represent the isolates used in this study, including genomes obtained from NCBI. Strains in bold represent the new genomes in this study and taxa representing ex-type cultures indicate with ^T^.


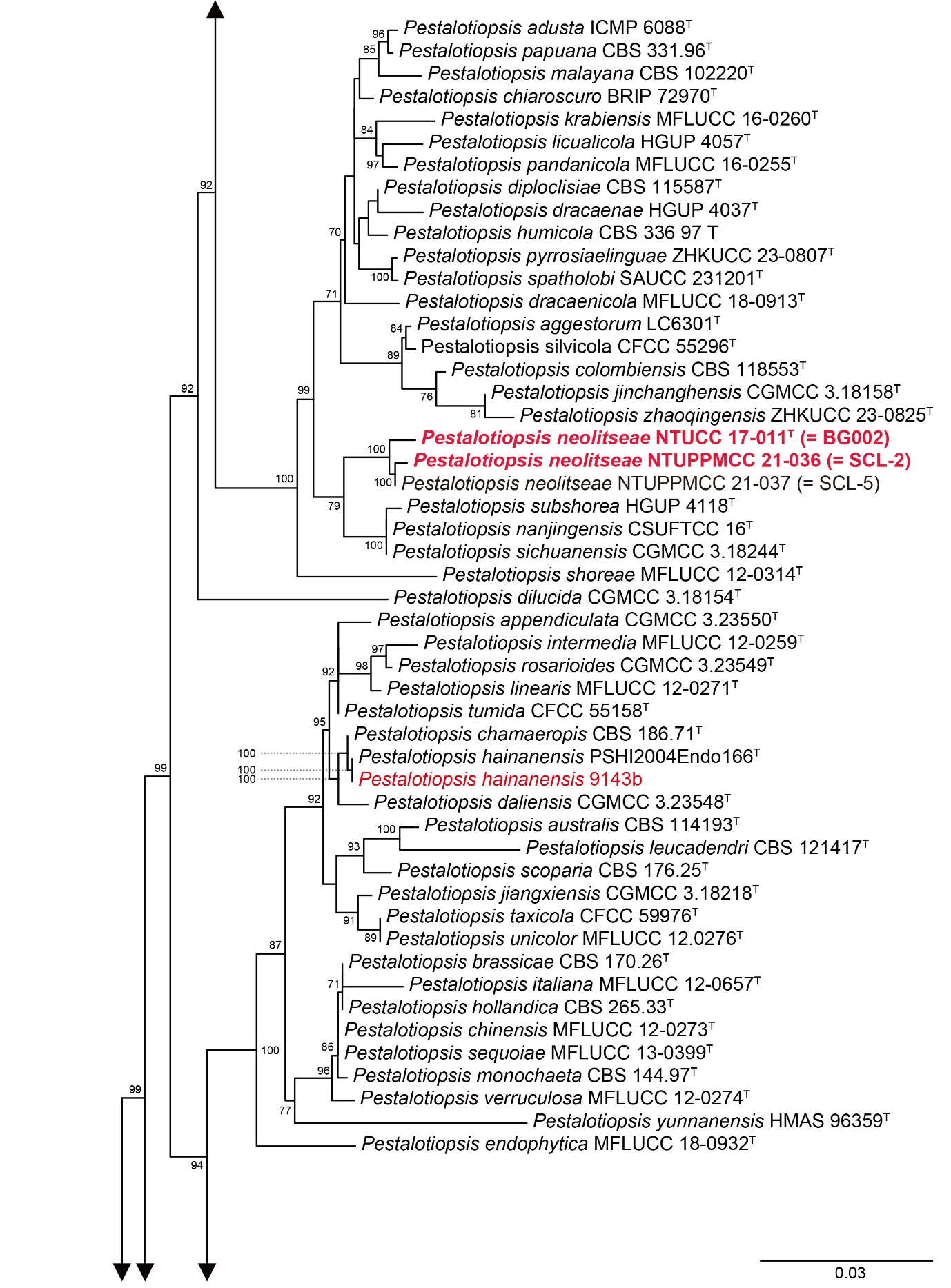


**Supplementary Figure S1. (Continued)**


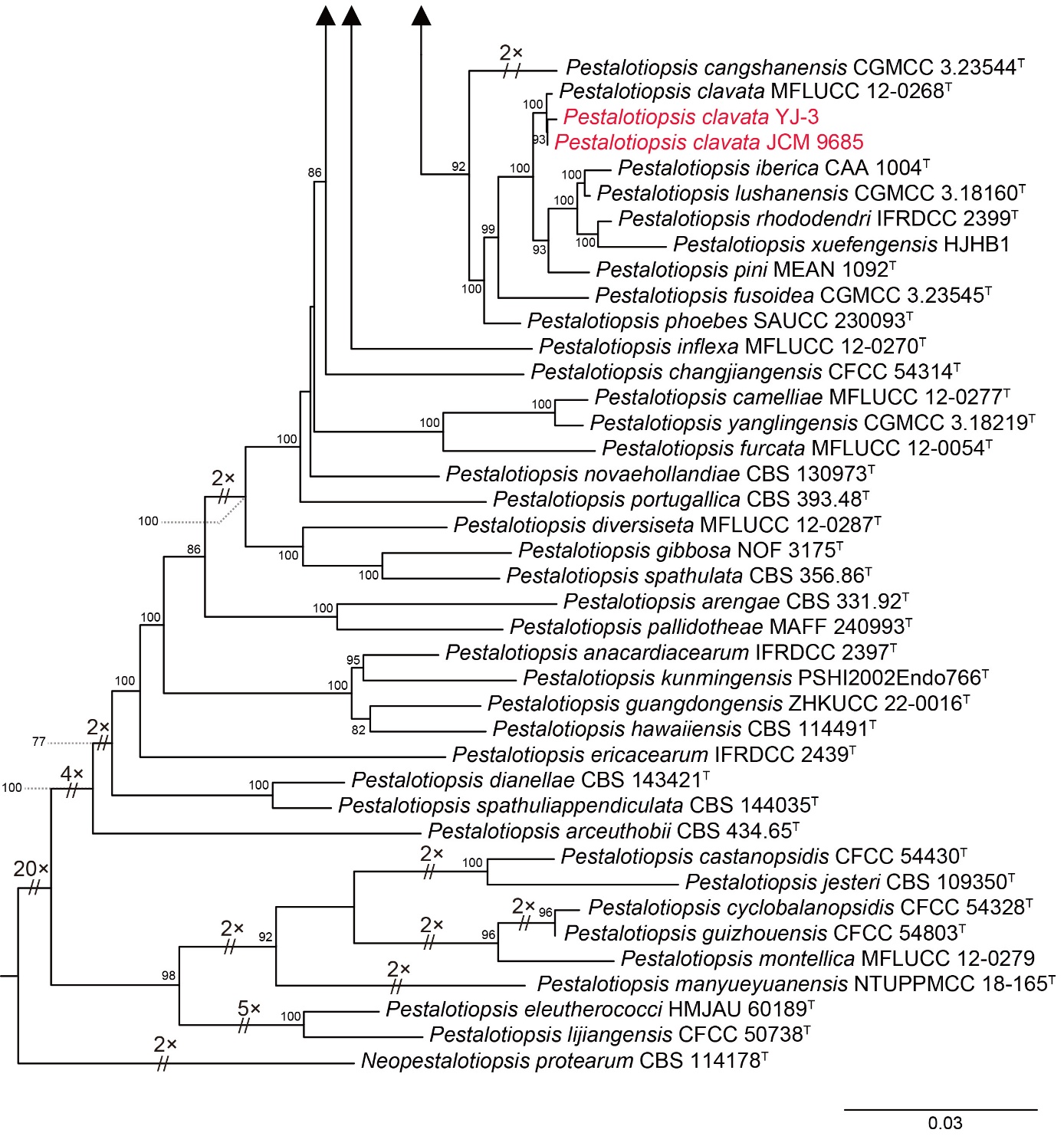


**Supplementary Figure S1. (Continued)**

**
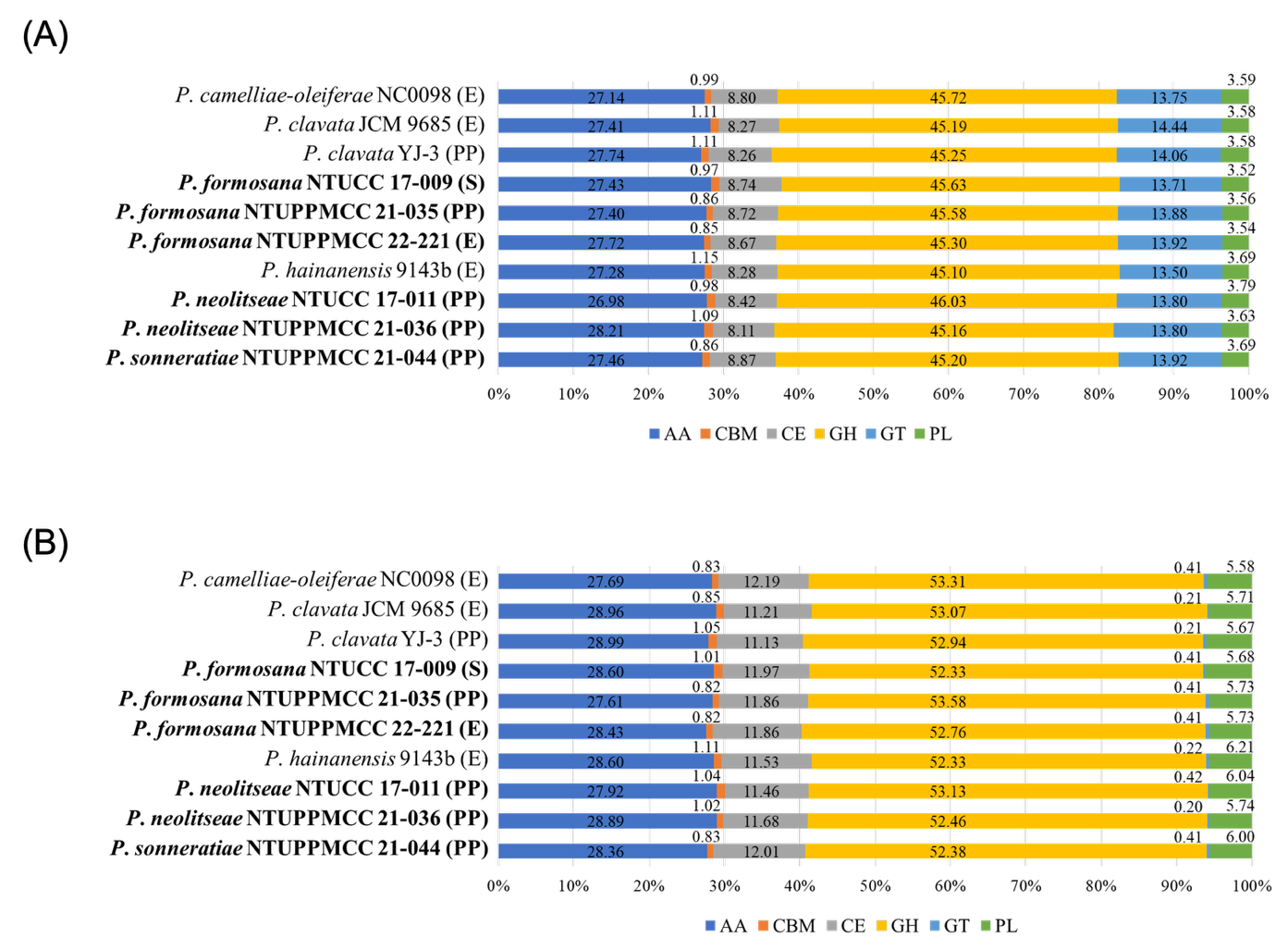
**

**Supplementary Figure S2.** (A) Ratios of CAZyme family composition in *Pestalotiopsis* strains analysed in this study. (B) Secreted-CAZyme family composition of *Pestalotiopsis* strains used in this study. The text in parentheses indicates the sampling context of each strain: E, isolate recovered from asymptomatic tissue; PP, isolate recovered from symptomatic tissue; S, isolate collected from dead or decaying material. CAZyme families are abbreviated as follows: AA, auxiliary activities; CBM, carbohydrate-binding modules; CE, carbohydrate esterases; GH, glycoside hydrolases; GT, glycosyltransferases; PL, polysaccharide lyases. Strains shown in bold indicate newly sequenced whole-genome assemblies generated in this study.


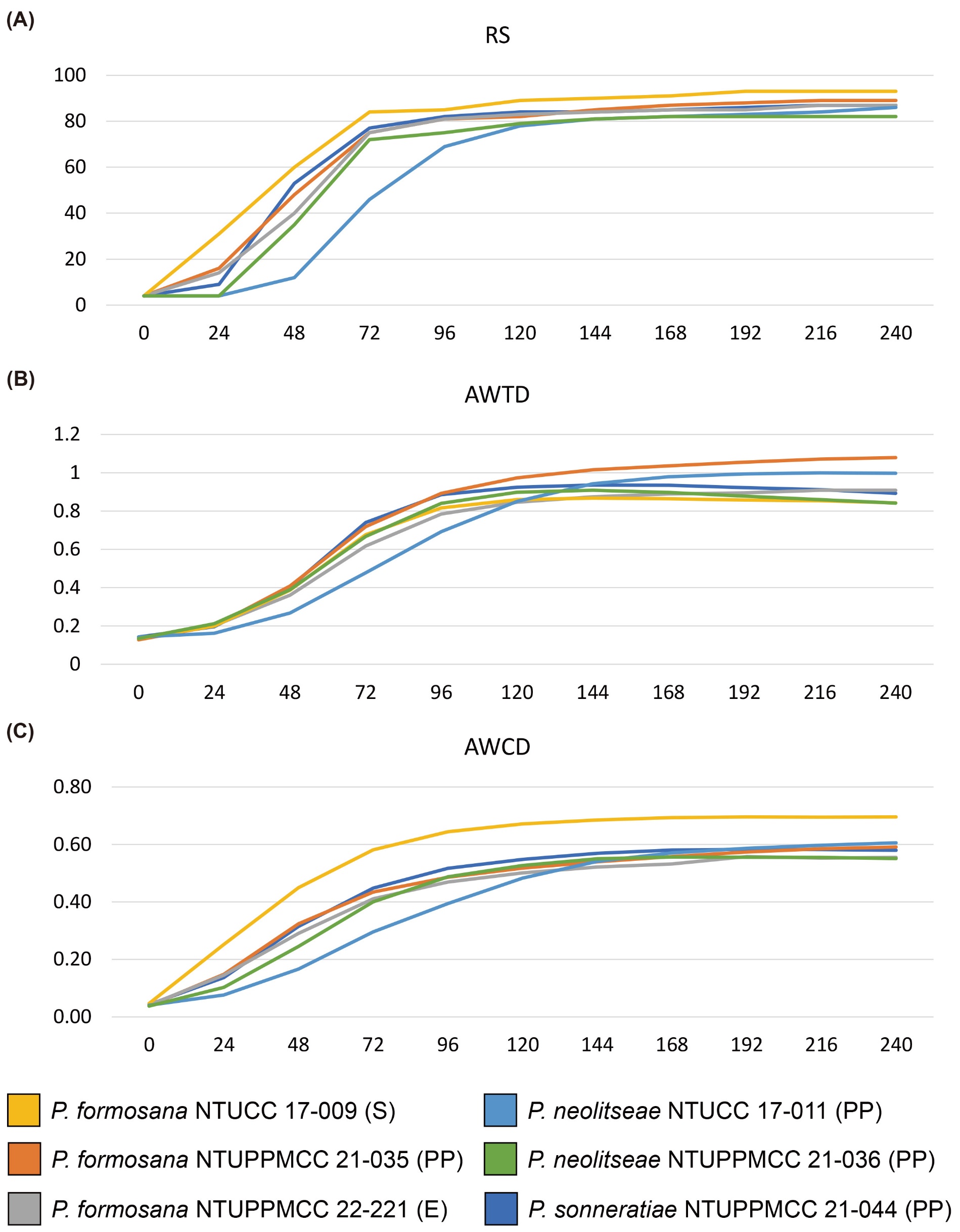


**Supplementary Figure S3.** Comparison of metabolic and growth characteristics of *Pestalotiopsis* strain on Biolog FF microplates. **(A)** Substrate Richness (RS), The number of substrates utilized by each strain over time. **(B)** Average Well Turbidity Development (AWTD), A measure of mycelium growth over time. **(C)** Average Well Color Development (AWCD), Reflects metabolic activity and substrate utilization. The text in parentheses indicates the lifestyle of each strain: E, isolate recovered from asymptomatic tissue; PP, isolate recovered from symptomatic tissue; S, isolate collected from dead or decaying material; X-axis: Time (hours).


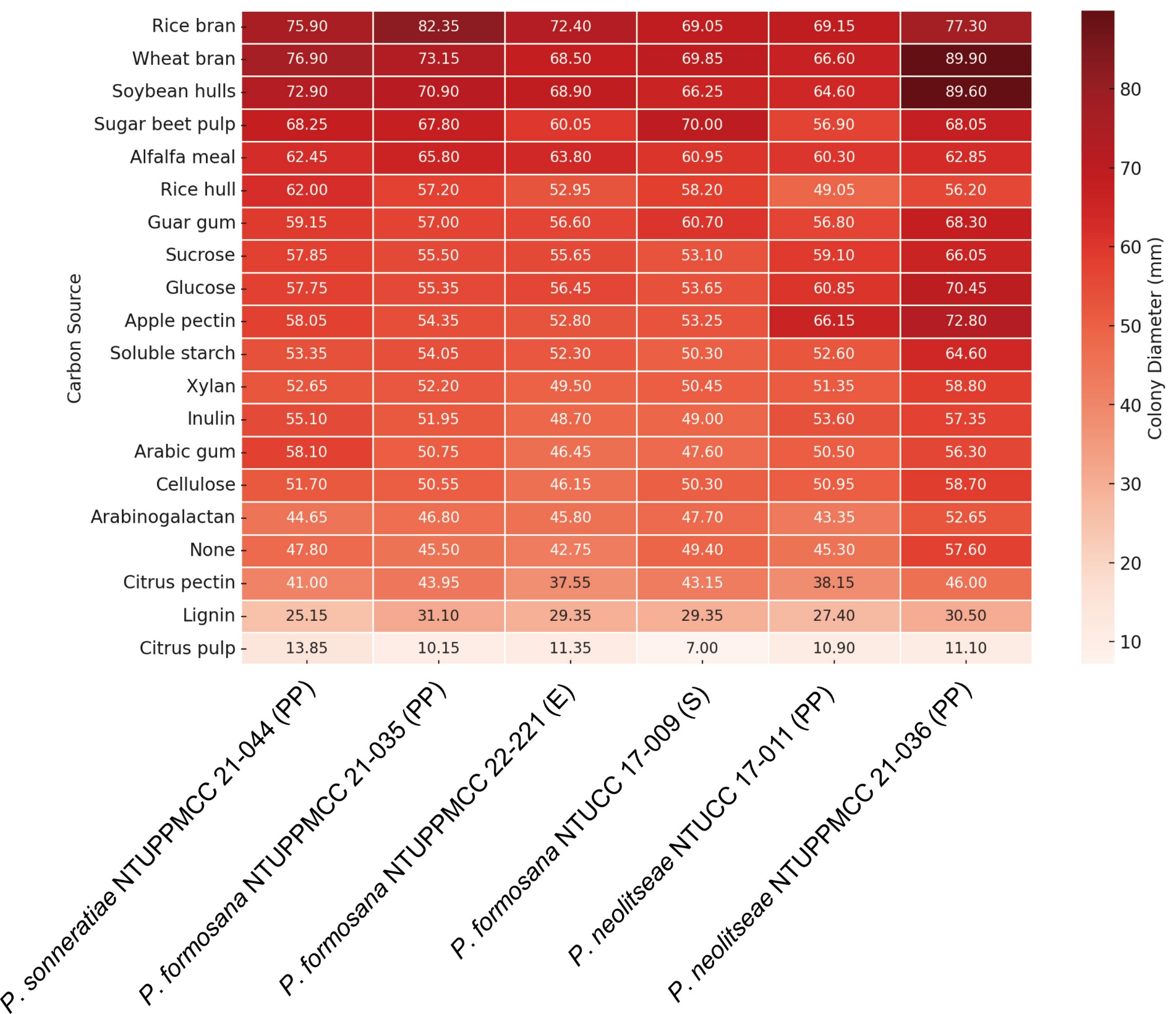


**Supplementary Figure S4.** Heatmap representing the colony diameter (mm) of *Pestalotiopsis* strains with different lifestyles grown on different carbon source media. The text in parentheses indicates the lifestyle of each strain: E, isolate recovered from asymptomatic tissue; PP, isolate recovered from symptomatic tissue; S, isolate collected from dead or decaying material.
